# Response to letter by Ulibarri and Bengelloun

**DOI:** 10.1017/S0007114522002276

**Published:** 2023-07-28

**Authors:** Marinos Elia, Rebecca J Stratton

**Affiliations:** Faculty of Medicine, University of Southampton, Mailpoint 113, Southampton General Hospital, Tremona Road, Southampton SO16 6YD, UK

Dear Editor,

It is somewhat surprising that Ulibarri and Bengelloun challenged the concept of MUST as a screening tool. Ever since its launch in 2003, MUST has been consistently referred to as a nutrition screening tool (test or instrument) for clinical practice by national and international, governmental and non-governmental organisations, as well as surveys and reviews on nutritional screening in clinical practice. It is also puzzling that Ulibarri and Bengelloun attempt to disqualify MUST as a screening tool based on a WHO definition that does not refer to screening in routine clinical practice, which *is* the remit of our paper. The part of the WHO definition that refers to asymptomatic populations just does not apply to the usual application of MUST or the populations in which it was validated. Ironically, Ulibarri and Bengelloun highlight their own screening work without mentioning that their screening was undertaken in a symptomatic hospitalised population burdened with disease and malnutrition^(1)^. They also do not mention that validation of their tool involved the use of symptoms, BMI and/or weight loss.

They cite two WHO publications to support their assertion that MUST is not a screening tool^(2,3)^. However, they fail to point out the fact that the first of these publications by Wilson and Jungner^(2)^ states this about a screening test: ‘It should be noted that, by definition, unrecognised symptomatic as well as pre-symptomatic disease is included’. The second publication^(3)^ refers to the Wilson and Jungner’s screening principles^(2)^ as ‘the gold standard in deciding on implementing screening programmes’. The second publication also specifically indicates that it is concerned with population-based screening in apparently healthy asymptomatic subjects, and it deliberately does not cover issues in clinical practice. Clearly, this second publication, and others based on the use of screening tests in apparently healthy asymptomatic subjects, does not apply to routine clinical practice^(4)^, which is what we were referring to in our paper. The second publication also may not apply to non-clinical settings, such as those involving the ageing general population or older free-living populations, since a high proportion of these are already symptomatic from one or more underlying conditions (with or without malnutrition) and/or the side effects of treatments. Not surprisingly, a recent WHO report ‘Screening programmes: a short guide’^(5)^ states ‘The technical definitions of screening and its subtypes are debated. This publication defines the terms but recognizes that these might not always align with the definitions that appear in other texts’. It goes on to emphasise that ‘Unfortunately, the terms used in this field are not consistent’.

From a routine clinical practice perspective, it seems unrealistic and inappropriate to screen for asymptomatic, protein-energy malnutrition because patients, with and without malnutrition in various care settings (e.g. in hospitals, outpatient clinics, general practitioner surgeries or in nursing homes) often have symptoms from underlying disease(s). Symptoms, such as tiredness, lethargy, weakness and fatigue, overlap with those observed in disease with and without malnutrition, as well as in malnutrition with and without disease. Malnutrition can be a cause or consequence of disease and *vice versa*. This is why MUST attempts to identify treatable malnutrition as early as possible and to prevent or attenuate deterioration.

All screening instruments have their limitations. Although laboratory tests, such as Ulibarri and Bengelloun suggest, can be useful, especially in investigating disease-related malnutrition, the commonly used nutrition screening tools in routine clinical practice, such as Nutritional Risk Screening-2002, short form Mini Nutritional Assessment, Simplified Nutritional Appetite Questionnaire and MUST, do not include laboratory tests. The reasons for this include: laboratory tests may not be readily accessible in some care settings; they often require expertise to draw blood and to perform immunological testing; and they can be costly and can cause delay in providing results. In contrast, MUST which can be used in a variety of care settings by healthcare professionals, healthcare assistants and members of the public (see MUST self-screening website; https://www.bapen.org.uk/screening-and-must/malnutrition-self-screening-tool) can generate results ‘on the spot’ and be incorporated prospectively into clinical management. Furthermore, certain laboratory markers of malnutrition may be misleading because they are non-specific and some of them, such as the circulating albumin concentration, are influenced more by the disease than directly by nutritional status (see MUST report for more discussion^(6)^). Further details about MUST, including the toolkit, its use in different languages and the MUST report can be found at https://www.bapen.org.uk/screening-and-must/must.
